# 肺腺癌组织中IGF-IR表达与患者临床病理因素及预后的相关性

**DOI:** 10.3779/j.issn.1009-3419.2010.12.06

**Published:** 2010-12-20

**Authors:** 雪艳 张, 久贤 冯, 惠芳 沙, 进肃 黄, 宝惠 韩

**Affiliations:** 1 200030 上海，上海交通大学附属胸科医院肺内科 Department of Pulmonary Medicine, Shanghai Chest Hospital, Shanghai Jiaotong University, Shanghai 200030, China; 2 200030 上海，上海交通大学附属胸科医院肺癌研究室 Basic Research Laboratory, Shanghai Chest Hospital, Shanghai Jiaotong University, Shanghai 200030, China

**Keywords:** 肺肿瘤, IGF-IR, 预后, Lung neoplasms, IGF Type 1, receptor, Prognosis

## Abstract

**背景与目的:**

肺腺癌发病率不断升高，而胰岛素样生长因子I受体（insulin-like growth factor I receptor, IGF-IR）是多种生长因子的调控枢纽，在肿瘤细胞的分化、增殖过程中起重要调节作用。本研究旨在为检测肺腺癌组织中IGF-IR表达，并分析其与肺腺癌患者临床病理因素及预后的相关性。

**方法:**

采用免疫组化方法检测肺腺癌组织IGF-IR表达。卡方检验分析IGF-IR表达与临床病理因素的关系，*Kaplan-Meier*生存曲线计算生存率，采用*Cox*分析评估各指标与患者生存期之间的关系。

**结果:**

126例肺腺癌组织中，89例可观察到IGF-IR阳性细胞。IGF-IR表达与肺腺癌患者肿块大小及T分期相关，而与年龄、性别、吸烟史、病理分期、分化及CEA等因素及患者的疗效及生存期无明显相关。

**结论:**

肺腺癌患者表达IGF-IR，与患者的肿块大小和T分期有关，而与预后无关。

## Abstract (en)

**Background and objective:**

The incidence of lung adenocarcinoma increases rapidly, and IGF-IR is the key mediator of several growth factors signal transduction, therefore it plays an important role in the proliferation and differentiation of cancer cell. The aim of this study is to detect the expression of IGF-IR in lung adenocarcinoma and to evaluate its implication for the clinicopathological factors and prognosis of patients with this disease.

**Methods:**

The IGF-IR expression was detected by immunohistochemical staining. Correlations between IGF-IR expression with clinicopathological factors were analyzed using the *Chi-squared* test. The *Kaplan-Meier* method was used to calculate the overall patient survival rate, and the difference in survival curves was evaluated using a *Log-rank* test. Univariate and multivariate analysis was carried out using the *Cox* proportional-hazard model.

**Results:**

In 126 cases of tumor sections tested, IGF-IR were detected in 89 cases. Statistical analysis revealed that the IGF-IR expression was related to tumor size and T stage, while there were no relations between IGF- IR expression and age, gender, smoking, pathological stages, and differentiation. *Cox* analysis indicated that metastasis and chemotherapy efficacy were the prognostic factors in these patients, while IGF-IR expression was not the independent prognostic factor.

**Conclusion:**

The IGF-IR expression is related to tumor size and T stage, while there is no relation between IGF-IR expression and prognosis.

近年来，肺癌发病率不断升高，而非小细胞肺癌（non-small cell lung cancer, NSCLC）占肺癌的80%。其中肺腺癌是NSCLC最主要的组织类型之一，其发病率不断升高，因而，对肺腺癌的细胞起源及癌变机制的研究受到国内外学者的高度关注。胰岛素样生长因子I受体（insulin-like growth factor I receptor, IGF-IR）是多种生长因子调控枢纽，在细胞生长、分化过程中起重要调节作用。越来越多的资料表明，IGF-IR在肿瘤组织中存在不同程度的异常表达，并在肿瘤细胞分化分裂、增殖凋亡中扮演重要角色。本研究旨在探讨IGF-IR在肺腺癌中的表达及其临床意义。

## 1 资料与方法

### 1.1 临床资料

收集上海交通大学附属胸科医院1999年1月-2004年6月经外科根治性手术切除且随访资料完整的126例肺腺癌患者纳入本研究。患者年龄22岁-80岁，中位年龄57岁；男性65例，女性61例。按国际抗癌联盟（International Union Against Cancer, UICC）标准进行分期，Ⅰb期30例，Ⅱ期39例，Ⅲa期57例。高分化30例，中分化76例，低分化20例。随访从手术之日开始，末次随访日为2009年12月15日，本组患者的随访时间均 > 5年。所有患者术前均未进行过放疗或化疗，术后接受2次-4次以铂类为主的化疗。手术标本经4%甲醛固定，石蜡包埋，制成4 μm厚切片。由两位病理科主任、副主任对原发病灶的病理切片进行审核，明确病理诊断。

### 1.2 免疫组化检测IGF-IR表达

免疫组化技术采用Supervision法，肺腺癌患者癌旁的正常肺组织作为阴性对照。具体操作步骤如下：石蜡切片60 ℃，烘片1 h-2 h；二甲苯脱蜡3次，每次20 min，梯度乙醇水化后蒸馏水冲洗3次；PBS冲洗，5 min×3次；二甲苯脱蜡后，用柠檬酸缓冲液pH 6.0（上海长岛生物技术有限公司）进行高温高压抗原修复，修复时间120 s；用PBS冲洗3次，每次3 min-5 min；切片滴加兔抗人IGF-IR单克隆抗体（DA-KO公司）50 μL，于4 ℃环境过夜；用PBS冲洗3次，每次3 min-5 min；滴加50 μL第二抗体Supervision（上海长岛生物技术有限公司）（按试剂盒使用），室温下孵育40 min；用PBS冲洗3次，每次3 min-5 min；每张切片滴两滴约100 μL的DAB显色液，显微镜下观察3 min-5 min；苏木精染色5 min，冲洗20 min；1%盐酸酒精分化，冲洗20 min；切片经95%、100%乙醇各两档脱水，放入电热恒温鼓风干燥箱风干；二甲苯透明，中性树脂封固。

### 1.3 免疫组化评定标准

免疫组化结果由两位病理科主任、副主任对切片进行审核。IGF-IR表达主要以细胞膜及胞质混合型表达为主，阳性染色呈棕黄色颗粒。评定用半定量方法：总的评定分数根据染色阳性细胞数的比率和染色强度。染色阳性细胞定量： < 5%为0分，5%-25%为1分，26%-50%为2分， > 50%为3分；染色强度：无染色为0分，染黄色为1分，染棕色为2分。最终的表达评定分数为：染色细胞定量分数×染色强度分数，分别为0分-1分为阴性（-），2分-3分为弱阳性（+），4分-6分为阳性（++）。

### 1.4 统计分析

实验数据应用SPSS 11.0统计软件进行分析处理。组间率的比较采用*χ*^2^检验。*Kaplan-Meier*方法计算生存曲线，用*Log-rank*进行差异检验。生存分析采用*Cox*单因素及多因素分析。以*P* < 0.05为差异有统计学意义。

## 2 结果

### 2.1 IGF-IR在肺腺癌组织中的表达

IGF-IR主要以细胞膜及胞质混合型表达为主，阳性染色呈棕黄色颗粒（图 1）；126例患者中89例表达阳性，其中18例（++），71例为（+）。阳性率为70.63%。

**1:**
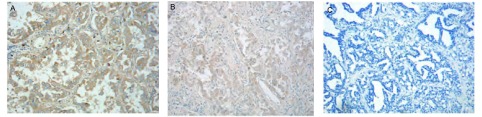
IGF-IR在肺腺癌组织中呈不同程度表达（免疫过氧化物酶染色，×200）。A：IGF-IR表达（++）；B：IGF-IR表达（+）；C：IGF-IR表达（-）。 Immunohistochemical staining of IGF-IR protein in lung adenocarcinoma tissues (immunoperoxidase stain, ×200). A: strong staining in the most of the lung adenocarcinoma cells (++); B: weak-moderate staining in lung adenocarcinoma cells (+); C: negative staining in lung adenocarcinoma cells (-).

### 2.2 IGF-IR表达与临床病理特征的关系

IGF-IR表达与肺腺癌患者肿块大小及T分期相关，与患者年龄、性别、吸烟史、肿瘤分化、病理分期及CEA等指标无关（*P* > 0.05）（表 1）。

**1:** IGF-IR表达与肺腺癌临床病理特征的相关性 Correlation between IGF-IR expression and clinicopathological factors in 126 patients with lung adenocarcinoma

Characteristic	IGF-IR		*P*
	(+)-(++)	(-)	
Age (years)			0.357
< 65	60	23	
≥65	29	14	
Gender			0.267
Male	48	17	
Female	41	|20	
Smoking status			0.513
No	62	26	
Yes	27	11	
Tumor size			0.022^*^
≤3 cm	27	19	
>3 cm	62	18	
Pathological stages			0.078
Ⅰb	19	11	
Ⅱ	24	15	
Ⅲa	46	11	
T stage			0.018^*^
1	3	6	
2	69	28	
3	17	3	
N stage			0.542
0	21	12	
1	32	13	
2	36	12	
Differentiation			0.843
Well	20	10	
Moderate	55	21	
Poor	14	6	
CEA			0.111
Normal	43	23	
Increase	46	14	
Statistical analyses were performed using Pearson *Chi-Square* test. ^*^P < 0.05.			

### 2.3 IGF-IR表达与转移和疗效的关系

126例患者中，95例发生转移，其中69例为IGF-IR阳性表达。85例患者可随访到转移后化疗疗效。IGF-IR表达与否与是否转移、转移途径、转移部位及转移后化疗疗效均无统计学差异（表 2）。

**2:** IGF-1R表达与肺腺癌患者的转移及疗效的相关性 Correlation between IGF-1R expression and metastasis and chemotherapy efficacy in 126 patients with lung adenocarcinoma

Characteristic	IGF-IR		*P*
	(+)-(++)	(-)	
Metastasis			0.260
Yes	69	26	
No	20	11	
Metastasis pathway			0.484
Lymph	14	6	
Blood	55	20	
Metastasis site			0.570
Lung	20	11	
Brain	13	2	
Bone	14	6	
Other parts	8	1	
Mediastinal lymphnode	9	4	
Supraclavicular lymphnode	5	2	
Chemotherapy efficacy			0.435
PR	4	0	
SD	33	14	
PD	25	9	
Statistical analyses were performed using *Pearson Chi-Square* test. PR: partial response; SD: stable disease; PD: progressive disease.			

### 2.4 IGF-IR表达与肺腺癌患者生存期的相关性

进一步分析IGF-IR和肺腺癌患者生存期的相关性，用*Log-rank*进行分析，结果表明，126例肺腺癌患者中，IGF-IR表达情况对生存期的影响无统计学差异。生存曲线趋势也表明，IGF-IR表达对患者生存无明显的影响（图 2）。

**2:**
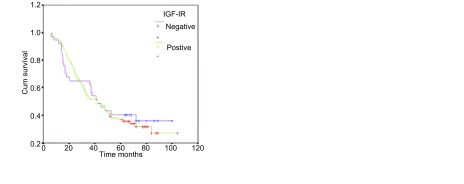
IGF-IR表达与肺腺癌生存期的关系（*Log - rank*=0.08, *P*=0.775） Cumulative *Kaplan-Meier* survival curves for patients with IGF-IR (*Log-rank*=0.08, *P*=0.775)

### 2.5 肺腺癌患者生存期影响因素的*Cox*回归模型分析

*Cox*比例风险回归模型对影响患者预后的多项因素进行单因素分析表明：年龄、病理分期、N分期、CE A、是否转移、转移后化疗疗效均影响患者的生存。进行多因素分析显示，是否转移、转移后化疗疗效均是影响生存期的独立因素。无转移的患者较有转移的患者生存期长（*P* < 0.001），相对危险度为0.005（95%CI: 0.001-0.043）。此外，转移后化疗效果好的患者比化疗效果不佳的患者预后好（*P* < 0.001），相对危险度为1.969（95%CI: 1.423-2.726）（表 3）。

**3:** 肺腺癌患者生存期影响因素的*Cox*回归模型分析结果 Univariate and multivariate analysis of clinicopathological factors for the overall survival rate of 126 patients with lung adenocarcinoma

Parameter	*β*	S.E.	Wald	Exp(B)	95%CI for Exp(B)		*P*
					Lower	Upper	
Univariate analysis							
Gender	0.024	0.220	0.012	1.025	0.666	1.577	0.912
Age	0.481	0.225	4.572	1.618	1.041	2.516	0.032*
Smoking status	0.104	0.220	0.222	1.109	0.721	1.708	0.637
Tumor size	-0.021	0.229	0.008	0.979	0.626	1.533	0.927
Pathological stage	0.346	0.145	5.673	1.414	1.063	1.879	0.017*
T stage	0.332	0.212	2.447	1.393	0.920	2.111	0.118
N stage	0.297	1.141	4.430	1.345	1.021	1.773	0.035*
Histological differentiation	0.086	0.189	0.209	1.090	0.753	1.578	0.647
CEA	0.900	0.227	15.708	2.460	1.576	3.839	0.000*
Metastasis	-4.210	1.014	17.236	0.015	0.002	0.108	0.000.
Chemotherapy efficacy	-0.274	0.113	5.841	0.760	0.609	0.949	0.016*
IGF-1R	-0.070	0.246	0.081	0.932	0.576	1.509	0.776
Multivariate analysis							
Metastasis	-5.233	1.059	24.399	0.005	0.001	0.043	0.000*
Chemotherapy efficacy	0.678	0.166	16.690	1.969	1.423	2.726	0.000*
Pathological stages	0.567	0.326	3.013	1.762	0.929	3.342	0.083
CEA	0.269	0.234	1.324	1.309	0.828	2.070	0.250
N	-0.419	0.326	1.653	0.658	0.347	1.246	0.199
Age	0.219	0.230	0.908	1.245	0.793	1.954	0.341
^*^P < 0.05							

## 3 讨论

IGF-IR是一种酪氨酸蛋白受体，它的主要作用是介导IGF-Ⅰ和IGF-Ⅱ的促生长活性。当配体与IGF-IR的亚单位中的结合部位结合后，激活位于胞内的酪氨酸激酶，引起胞内信号转导，进一步引起细胞分裂分化和组织器官的生长发育。细胞的正常增殖分化和机体的正常发育均受IGF-IR介导的各信号传导途径的调节，当IGF-IR介导的信号传导失常时，机体可能形成肿瘤。IGF-IR在肿瘤的发生发展过程中起着多方面的作用，如促进细胞向恶性表型转化、促进肿瘤细胞生长和分裂增殖、抑制肿瘤细胞凋亡并与肿瘤的浸润有密切关系。研究发现IGF-IR在肺癌、肝癌、前列腺癌中呈过度表达。

本研究发现，IGF-IR表达与患者肿块大小和T分期相关，病灶大及T分期晚的患者IGF-IR表达增高。其可能的机制为：IGF-IR既可以结合IGF-Ⅰ，又与IGF-Ⅱ具有很强的亲和力，在IGF信息传导通路中发挥重要作用。正常情况下，细胞表面的IGF-IR对细胞凋亡无明显的影响。细胞癌变时，各种癌基因和抑癌基因的突变及相互作用而致肿瘤细胞中*IGF-IR*基因表达异常。表达IGF-IR的肿瘤细胞通过合成和分泌内生性IGFs，借助IGFs/IGF-IR环路刺激肿瘤细胞无限增殖，并维持其恶性表型。IGF-IR的过度表达可以阻止肺癌等肿瘤细胞的凋亡，且与肿瘤的浸润性生长、转移关系密切。在实验研究中发现，通过抗IGF-IR抗体、IGF-Ⅰ类似物或反义RNA使IGF-IR功能失活或数目减少，均可导致相应肿瘤细胞系大批凋亡，阻止体外增殖，并使其在同源动物或裸鼠体内的致瘤性丧失，可见，IGF-IR在建立和维持肺肿瘤转化表型中扮演着重要角色，可以成为抑制细胞增殖的合适靶点。

本研究也发现，IGF-IR表达与患者肿块大小和T分期相关，而与患者年龄、性别、吸烟史、肿瘤分化、病理分期、远道转移及CEA等指标均无关，且与是否转移、转移途径、转移部位及转移后化疗疗效均无关。生存曲线趋势也表明，IGF-IR表达对患者生存无明显的影响。因此，IGF-IR可能并非有效的预测肺腺癌疗效及预后的标志。近年研究^[1, 2]^发现，IGF-IR在乳腺癌中表达与疾病进展及放疗耐药相关，且预示不良预后。对IGF-IR表达与临床指标及预后的关系，各家报道不一致，目前仍有争论。有关前列腺癌和乳腺癌的研究^[3, 4]^显示，早期肿瘤和正常组织中IGF-IR表达增多，而进展性癌表达减少。IGF-IR表达提示预后良好，而在软组织肉瘤的研究中也支持这一观点^[5]^。而也有相反的观点认为，IGF-IR揭示不良的预后^[6, 7]^。对肺癌的研究较少，Merrick等^[8]^的研究表明，IGF-Ⅰ R在肺腺癌中表达高于其它组织类型，有统计学意义，且与不良预后有关。而Lee等^[9]^对71例Ⅰ期NSCLC的研究表明，IGF-IR表达与临床指标（如性别、组织类型、分期、分化等）无相关性，且和患者预后无相关。而最近Ludovini等^[10]^研究发现VEGF和IGF-IR共表达的患者预后不良，而单独IGF-IR表达和预后无关。最近的一项研究^[11]^通过对189例NSCLC的手术标本进行检测IGF-IR，发现IGF-IR表达与EGFR表达具有相关性，且IGF-IR蛋白表达与肺癌患者分期有关，但与肺癌患者的生存无关。因此，目前对IGF-IR对肺癌患者预后及临床指标关系的观点说法不一，本研究显示，IGF-IR表达与肺腺癌转移及预后等无关。

*Cox*单因素和多因素分析表明：年龄、病理分期、N分期、CEA、转移及转移后化疗疗效均是患者生存期的影响因素。高龄患者易进展，这可能与老年患者心肺功能相对较差，以及对手术的耐受性不如年轻患者好等因素有关；CEA也与病情进展有关，而多因素分析表明，是否发生转移及转移后疗效均是影响生存期的独立因素。患者出现转移后，依据转移部位，临床医生所选择的治疗方法不同，包括化疗、放疗、再次手术等。而且转移的部位不同，化疗方案也不尽相同，如DP、D、Me- ccnu+VM26、GP、TP等。由于方案种类较多，且每种病例数较少，故未进行化疗方案与预后关系的分析。共有85例可随访至有化疗疗效，分析疗效与预后的关系发现二线化疗疗效为预后的独立影响因素，而IGF-IR并非影响患者预后的独立因素。因此，IGF-IR与肿瘤的形成、侵袭、肿瘤细胞增殖等相关，可能成为抑制细胞增殖的作用靶点，但并不适合作为预测疗效及预后的适合指标。
